# Decentering globalization: processes, conditions, and worldviews

**DOI:** 10.3389/fsoc.2026.1941732

**Published:** 2026-10-29

**Authors:** Victor Roudometof

**Affiliations:** Department of Social and Political Sciences, University of Cyprus, Nicosia, Cyprus

**Keywords:** culture, globalization, glocalization, localization, sociological theory-general

## Abstract

This article argues that the notion of “globalization” that was popularized after communism’s collapse has become empirically defunct and intellectually indefensible. To decenter globalization, this article argues in favor of revising our understanding of the “global” beyond global modernization or the modernization of the “world.” The article proposes to pluralize the concept and adopt a trinity of terms: “global,” “glocal,” and “local.” This solution differentiates the substantive dimensions implicit in most analyses of the “global.” Each of these terms should be seen as analytically autonomous from the others; taken together, they form a trinity of mutually defined concepts. An overview of these concepts is presented, whereby specific terminology for each distinguishes among the processes signified, the conditions indicated, and the worldviews that express normative endorsement of each concept.

## 1 Introduction

While “decentering globalization” (or the “world”) is a phrase that has been used in literary criticism, post-colonial studies and international relations, no systematic account of it exists in the sociological literature. This article challenges the centrality of globalization (or global modernization) as the main concept that underpins a master narrative of social change. It decenters the paradigm of global modernity by altering its components. The first section lays out the rationale for replacing the notion of globalization as global modernization^1^. Next, the discussion shifts to the requirements for such decentering. To replace the dominant version of “globalization,” a tripartite cluster of concepts is introduced. These are analyzed in terms of processes (globalization, glocalization, and localization), conditions (globality, glocality, and locality), and worldviews (globalism, glocalism, and localism). Alongside the theoretical expose, a few examples are used for illustration. Regardless, this explicitly programmatic or heuristic outline does not include an empirical component. It operates from within the lenses of cultural sociology; and focuses on meaning-contexts instead of power asymmetries or domination.

This paper is limited to the problematic of globalization. It does not extend into the related notion of transnationality. Reflecting the regional unevenness of past scholarly output, much of the scholarship surveyed comes from the North. However, non-Western concepts^2^ and scholarship from the South & by non-Western scholars are set in dialogue with Western accounts^3^.

## 2 Globalization: the word beyond the “world”

Globalization has been simultaneously used both as a “first order” and a “second order” concept (to use Schutz’s (1962) classic distinction). In 20th century phenomenology, “first order” concepts are those used by the social actors themselves; these are terms used in everyday language by laypersons. In contrast, “second order” concepts are those devised by analysts to make sense of the social universe as experienced by the actors. During the 1990s, the “first order” notion of globalization saturated public policy debates. By the 21st century, this interpretation (often seen as neoliberal) had become passé.

Unlike its “first order” understanding, in academia, “globalization” was an early shorthand for “global modernization” or the “modernization of the whole world” (Robertson, 2014a). Neither “global” nor the “world” are self-evident or naturally existing concepts (Conrad, 2017: 185). Global modernization intertwines “globe” and “world” (or “lifeworld” as 20th century phenomenology used to refer to it). The “world” of modernity extends to the entire globe, as in Robertson’s (1992: p. 8) definition of globalization, as the process whereby “the world becomes a single place.” This phenomenological or metaphorical understanding of the “world” has a long history. Diverse cultures have developed various understandings of their “world,” ranging from Pythagoras’ *cosmos* to the *ecumene* of the Roman Empire to the Chinese notion of *tianxia* (Wang, 2012; Webel, 2021)^4^.

Whether singular or pluralized, this notion of the “world” assumes the existence of a level of internal cohesion within each individual “world.”^5^ That, in turn, might be more imagined than real. Consider, for example, the sheer volume of scenarios or scripts about global or world culture. These include the Americanization and/or Westernization theses, which claim the “world” is a replica of US or Western societies (Beck et al., 2003; Ritzer, 2003); the “glocalization” (Robertson, 1995; Roudometof, 2016) or hybridization (Bhabha, 1994) theses, which suggest that the “world” is characterized by a fusion between Western and non-Western cultures; and the “clash of civilizations” thesis (Huntington, 1996), which proposes that global cultural warfare could occur between proponents of rival cultural orientations (“civilizations”). In practice, these scenarios operate in part as rival master narratives of social change. When that is the case, it is assumed that worldwide trends coalesce along the path, or the trajectory proposed by each rival interpretations.

More broadly, the various instances and thematic cases of globalization across social-scientific literature do not appear to offer unambiguous support for any of the narratives currently in circulation (Pieterse, 2021: p. 71). To argue in favor of the universal reach of either of these (or any other) narratives presupposes “a genuinely global integration [that] has never occurred, and presumably never will” (Conrad, 2017: pp. 102–103)^6^.

The above reinforce the necessity to question their shared underlying assumption: that it is possible and/or meaningful to construct a single uniform Grand Theory about the “world,” or to put it differently, that the “world” (either in its singular or plural version) provides sufficient conceptual foundations^7^. Globalization has been applied to numerous areas and topics; with timelines that generally do not align (overview in Pieterse, 2012). This temporal misalignment is a consequence of the way globalization is seen by scholarly and lay communities. Resolving this conundrum requires re-examining the concept. Globalization involves a shift into spatial logic, conventionally framed in terms of a dialectical relationship between deterritorialization and reterritorialization (Held et al., 1999; Scholte, 2019). While old territorial attachments are decoupled, new forms of such attachments are forged through re-territorialization. Held et al. (1999: p. 16) operationalize globalization as a set of processes which embody “a transformation in the spatial organization of social relations and transactions.” This transformation creates new spatial formations forged through transcontinental or interregional flows and networks; and these affect power relations globally.

For Held et al. (1999), extensity, intensity, velocity and impact are measurements of globalization’s impact. The scaling of these indicators implies that global social integration might be the *telos* of globalization. The notion of “de-globalization” was subsequently introduced to capture opposing trends (Bello, 2002) and soon pro- and anti- trends formed a conceptual binary of options within a single trajectory (see Held and McGrew, 2007). But global social integration has been questioned; and more complex alternatives have been suggested. Meyer (2007), for example, has proposed that globalization might be seen as “exchange”, that is, the “expanded interdependencies and rates of transaction around the world” Meyer (2007, p. 262); as well as “a very widespread cultural consciousness […] of interdependence and of local and national embeddedness in world society” Meyer (2007, p. 263). Globalization might be wave-like (as suggested by Therborn, 2000), an interpretation that insinuates a circular relation between globalization and localization. From within historical scholarship, Conrad (2017: p. 6) has connected global history to “integration,” whereby patterns of exchange are “regular and sustained, and thus able to shape societies in profound ways.” But Conrad rejects the idea that “integration” refers to systemic properties and connects this notion to effect of the “global” as a factor or context for local action. Conrad’s “integration” resembles what in sociological jargon is usually referred to as global connectivity (Pieterse, 2021).

To make sense of these different options, it might be useful to think of globalization as having the potential to operate both as interactive and integral (Roudometof, 2020). While integral globalization brings forth new empirical objects and realities, interactive globalization might concern solely “exchanges” between actors and units; that is, the units that participate in such exchanges might be influenced by global cultural influences in ways that do not in fact align with the projected *telos* of global social integration. After all, cultural diffusion is effective insofar as the adopters have strong relations to the cultural models adopted. In the absence of strong relations, diffusion brings forth innovation (Strang and Meyer, 1993; Gluck and Tsing, 2009). To address this issue, scholarship has sometimes resorted to the awkward expression “globalization/glocalization” to denote both the movement toward global integration as well as the glocalized hybridity produced (see, for example, Beyer, 2021).

However, scholarly and institutional developments have strongly favored the overall supremacy of the notion of global. Specifically, from the late 1990s forward, Global Studies has been constructed partly around the claim that globalization is the central concept (Steger and Wahlrab, 2016; Robertson, 1990). Thus, when global trends began to reverse, scholarship has interpreted such developments in terms of “de-globalization” and/or “re-globalization”—for these were the only viable alternatives that could preserve the master concept’s centrality (Steger et al., 2023; Steger and James, 2019).

The extent to which this interpretation is persuasive for the broader scholarly community is debatable. Some have insinuated that the entire vocabulary might be inadequate to the challenges of the present; for example, the group of intellectuals and academics who form The Global (De-)Centre (2026) have stated in their Manifesto: “The fluid 1990s global conceptual lexicon to which many of us contributed (including expectations of flows, networks, transnationalism, and cosmopolitanism) is out-of-sync with current realities.” In perhaps the most extreme position, Escobar (2019) proclaims that the “global” *as such* does not exist. These interpretations reflect the necessity to question the idea of global social integration that was introduced in the post-1989 world. In such debates, the divergences between the “first order” and the “second order” notions of globalization are often set aside. In contrast to what is often asserted or merely assumed, “one-worldism” used to be a notion once popular among business authors or journalists and most sociological perspectives are different from this vision.

## 3 Decentering: from singularity to a trinity of terms

To the extent that global modernization is guilty of methodological nationalism and Eurocentrism (as suggested by Conrad, 2017: pp. 3-4), its shortcomings become self-evident. If the concept of globalization is defined as or tied exclusively to global modernization, it remains a legitimate target for scholarly criticism (Roudometof, 2024). However, it is no longer the case that globalization is intimately connected to global modernization, as it was in its early days. Since the 1990s, its use has exploded both in the social scientific literature as well as in the popular press (as a “first order” concept)^8^. Through the term’s wide proliferation, its initial connection to “the world” has receded as journalists, laymen, and scholars use the term in a general sense. This development is a double-edged sword: On the one hand, the voluminous literature generated since the 1990s has made globalization a key term in the vocabulary of lay and scholarly communities; on the other hand, its conceptual slippage into a general term means it now requires reframing.

To effectively accomplish this reframing involves redefining and disentangling the concept from its past articulation within the master narrative of global modernization. As Darian-Smith and McCarthy (2017: 8) remind us, to decenter means to disconnect from practical or theoretical assumptions of origin, priority, or essence. To decenter globalization means to disconnect the term from its assumptions and/or origins, or from the connotations initially ascribed to it when placed in the grand narrative of the West’s global modernization. This narrative goes back to the Cold War, when Lipset (1960) pointed out the correlation between greater levels of GDP and Western forms of democratic government. In the eyes of the West, successful global modernization would increase pro-Western democratic regimes. Unlike the Cold War era, in the 21st century a growing share of global economic activity belongs to BRICS+ (formerly known as BRICS) (see data in Sachs, 2024). The post-1989 success of the capitalist West over the communist East was characterized as a triumph of global neo-liberalism and the public policy project of “globalization,” which suggested neo-liberal economic policies as a catch-all formula. Arguing against the post-1989 global neo-liberal narrative, numerous critics have deployed post-colonial or neo-Marxist viewpoints to legitimately critique the concept, often without sufficiently differentiating between its “first-order” and “second-order” versions. As a result, it has become commonplace to point out how globalization has hindered, rather than helped the South.

However, to reject globalization as a scholarly (“second-order”) concept is far from identical to rejecting the popular (“first-order”) notion of globalization. To decenter globalization means to think of it outside the confines of the defunct narrative of global modernization and/or that of global neo-liberalism. Conrad (2017: p. 3) argues that “the social sciences are no longer adequately able to pose the right questions and generate answers that help explain the realities of a networked and globalized world.” But that is an exaggeration. Scholarship has realized this necessity, and different ideas have been in circulation. By the 21st century, a multitude of alternatives emerged to the great narrative of Western modernity and/or of global modernization. While an exhaustive list might be impossible to compile, some of these proposals include multi-polar globalization (Pieterse, 2018); a renewed vision of globalization (Steger and James, 2019); a revival of localism (Livesey, 2017); or the “refiguration” of geopolitical governance across multiple spatial topographies (Knoblauch and Löw, 2024).

When the notion of global is disentangled from global modernization, it can be viewed as a general meaning-context through which to interpret human affairs. In favor of this far more flexible interpretation is scholarly research that goes beyond modernity into older or pre-modern historical eras, or even into archeology (see Hodos, 2017; Khondker, 2025; Roudometof, 2014). The employment of a global template for understanding human affairs turns Western modernity and the “rise of the West” (McNeil, 1963) into phenomena that take place *within* the broader context of world-historical globalization (Hobson, 2004; Sterns, 2010). As a result, Western modernity no longer appears as parthenogenesis or the consequence of a linear progressive development that privileges Eurocentric historical accounts. Instead, scholarship is empowered to critically examine the interconnections among different world regions even beyond conventional temporal frameworks. This approach aligns with Bhambra’s (2016: p. 962) critique of privileging of internal factors in the rise of the post-1492 transatlantic West. It accepts the necessity to incorporate the connections between “Europe” (e.g., Western Europe) or the (transatlantic) West and the rest of the world.

Decentering globalization means that the hitherto polysemous and ambivalent concept of global modernization is superseded by a trinity of terms: “global,” “glocal,” and “local.” These terms have been in circulation for decades. But the prevailing academic wisdom subordinates glocal and local to the global or views them as derivative byproducts—that is, as secondary processes that can be easily cast aside (more in Roudometof, 2016). However, as the next section shows, concept pluralization offers a far clearer reflection of reality. This solution implements Pieterse’s (2018: p. 182) suggestion that, in our own era, “theories and concepts become plural in a fundamental sense,” for the singular is appropriate to a bygone era of Western universalism. Concept pluralization reflects the growing realization that, in the 21st century, globalization might not necessarily be “the central concept” (as Robertson, 1990 argued) but instead merely just one among a plurality of concepts (inclusive of “transnational,” “cosmopolitan,” etc.) that, during the second half of the 20th century, have coalesced into a new conceptual vocabulary that has reshaped the social sciences at large (further discussion in (Roudometof, 2021).

Indeed, the conceptual strategy of turning any specific term into a master concept fails to abide by the standard scientific practice of using concepts in a bounded manner. When a concept is overextended, it is rendered vacuous & trivialized. Globalization has been overused, thereby leading to murkiness, both theoretical and empirical. Concept pluralization is a means for addressing the lurking ambiguity that has hindered the globalization problematic—and opens the theoretical space to perform the decoupling that is implicit within the notion of decentering. Unlike most scholarly engagements, the suggestion is that none of these concepts (global, glocal and local) should be seen as possessing a superior meta-theoretical or normative status vis-à-vis the others^9^. This trinity of terms does not render itself into a new master narrative that might deliver some sort of mystical ontological security—instead the approach outlined here echoes Tsing’s (2015: p. 20) remark that our “precarious world is a world without teleology”.

## 4 Processes, conditions, and worldviews

To sum up the argument set forth thus far, thanks to the gradual refinement and explorations of the notion of “global” by hundreds (perhaps thousands) of researchers post 1989, the collective wisdom makes it possible to develop an interrelated taxonomy of three distinct concepts (“local,” “global,” and “glocal”). This section offers an outline of the proposed trinity of terms “global,” “glocal” and “local.” Each of these can be (and has been) used to designate a process (−zation), a condition (−ty), and a worldview or ideology (−ism). This classification follows scholarly practice and mostly reflects the manner this terminology is used in the social sciences—at least to the best of the author’s understanding.

The objective is not to offer a terminological exegesis per se, but instead to relate these concepts to each other and explicate how these contribute to decentering the previously dominant version of globalization. After all, terminology is not an end in itself; terms are useful because they help us grasp important aspects of reality. While this trinity of terms features the full spectrum of possible options, the entire spectrum is not necessarily equally applicable to every single empirical instance. In contrast, taken as a whole, these options form a toolkit for researchers. The relevance and suitability of each term becomes a matter to be decided by individual scholars who are familiar with the specifics of different empirical instances.

The tripartite classificatory framework is presented in Table 1.

**Table 1:** Global, local, glocal: processes, conditions, worldviews.

Process	Condition	Worldview
Globalization	Globality	Globalism
When *Z* (whatever *Z* might be) becomes worldwide, planetary, or interregional, inclusive of approximately or elliptically becoming so	*Z* is experienced in the form of a singularity throughout the planet, inclusive of synchronicity across the globe	“One-worldism,” the view of global social integration as normatively desirable, inclusive of public policy that surrenders local priorities and needs to global trends
Glocalization	Glocality	Glocalism
The refraction of global influence into a locality, creating fusions between global and local	The experience of the global that considers local cultural specificity, as captured in cultural *pastiche*	The advocacy of pragmatism to bridge the gap between global imperatives to local demands and needs
Localization	Locality	Localism
The transformation of a physical location into a place, crafting cultural distinctiveness through reflexive practices	The condition of a locale experienced as a place, i.e., a distinct or unique cultural environment	The advocacy of public policy strategies that subordinate global political and economic imperatives to local demands

### 4.1 The processes

Given that globalization is applicable to numerous processes, conditions, artifacts, cases, or other facets of human existence, *Z* stands for whatever the specific site or case might refer to. As elsewhere suggested (Roudometof, 2020), globalization might be (re-)defined as a process whereby *Z* (whatever *Z* might refer to) becomes global, worldwide, or interregional (or even merely regional, at least according to some authors). In this manner, the concept connects to a multitude of instances, fields, and thematic areas of interest without necessarily having to revert to a totality (such as “the world”) and without an implicit teleology.

This idea stands in sharp contrast to Robertson’s (2014b: p. 8) argument that “globality and locality are relative terms.” It is worth sketching, however briefly, the underlying intellectual dilemma and the reasons for introducing this revised definition. In Robertson’s (1992, 1995) initial interpretations of globalization and glocalization, the former turns into the latter. It thus bridges the gap between global and local. This reading of “globalization as glocalization” analytically conflates the two concepts (further discussion in Roudometof, 2016: p. 45–62). By privileging the global as his “central concept,” Robertson (1990) delegated the notions of local and glocal to the status of residual categories. Such residual categories result from efforts to achieve *post hoc* coherence to an interpretation facing empirical and/or theoretical challenges (see Alexander, 1988). Residual categories are a means for addressing empirical evidence that calls the initial interpretation in question without addressing underlying theoretical anomalies.

Robertson’s interpretation has been criticized over its failure to resolve the global–local opposition. As Connell (2007: 374) writes, Robertson’s reading of glocalization merely asserts “both terms of a static polarity at once,” and as a result, under the guise of various forms (either as local versus global or national versus global), this “duality continues to structure both debate and research.” Radhakrishnan (2010: p. 27) has expressed this criticism more concretely: “How are “local” and “global” cultures to be identified as analytically separate if they are completely enmeshed in one another?” As argued above, when it comes to culture, empirical evidence suggests that the singularity of meaning often imagined as an inevitable consequence or *telos* of globalization is not realized in practice. Robertson’s (2020) eventual endorsement of glocal as the appropriate description of the overreaching process might be viewed as an indirect response to this criticism; but the broader problem of treating glocal and local as residual categories remains unresolved.

In contrast, from the point of view advanced here, globalization ought to be applied *exclusively* to those instances in which phenomena, practices, ideas, models, or a specific theme or domain of action (*Z*) spreads throughout the globe or comes reasonably close to it. As Marling (2006) has shown, concrete things that are globalized are often technical, suggesting that local actors operate pragmatically. The majority of cultural items though rarely fall into this category, as locals in different places tend to apply their own interpretation to ideas & blueprints that have come from elsewhere to suit their own tastes. When words cross cultural boundaries (together with the ideas they carry), cross-cultural borrowing creates new “things” (see examples in Gluck and Tsing, 2009).

This revised generic definition of globalization facilitates the realization that the difference between global, on the one hand, and glocal and local, on the other hand, is consequential—numerous instances exist where the former is used instead of the latter. This paves the way for recognizing the autonomy of glocalization and localization as distinct processes. As elsewhere suggested (Roudometof, 2016: p. 62–79), glocalization is globalization refracted through the local. This means that glocalities are generated through processes of interactive globalization, whereby the local is not annihilated by the global. Upon closer scrutiny, numerous instances where globalization has been conventionally evoked turn out to be instances of glocalization (see Roudometof and Dessi, 2022 for an overview of the state of the art): K-pop, theme parks (from Shanghai to Florida), religious organizations, immigrant communities, sports, and wine production have been used as sites for the glocalization of practices, goods, or activities. Additionally, even processes that seem indisputably global (such as climate change) possess a glocal dimension (see Haller et al., 2019). Even the COVID-19 pandemic illustrated the relevance of glocal health strategies in addressing a global health hazard. This omnipresence and/or the very banality of glocalization in contemporary life leads laypersons to misinterpret glocal expressions as local: K-pop, for example, is not “authentic” Korean culture, but a fusion of global pop recipes and local Korean features (see Oh, 2017).

But interactive glocalization does not obliterate the local. As Roudometof (2019) has argued, to distinguish localization from glocalization it is best to conceive of the local as a “place” (in the sense this term has for humanist geographers). The notion of “place” is the outcome of making geographical location meaningful through the imputation of emotions, connections, and human action. Localization then becomes a process whereby cultural items are identified with a specific place and gain meaning from this association. Localization emerges as a corollary of globalization itself: localization is place-making at the local level. It is *not* a process of mere adaptation of global imperatives to local situations (e.g., “top-bottom” processes) or a reactive backlash against globalization. Recovering the local is of central significance for understanding the world around us (full discussion in Roudometof, 2019). As Kennedy (2017) argues, the everyday lives of most people in the world remain dependent upon interpersonal relations and local attachments (see also Trundle and Bönisch-Brednich, 2010). Echoing this realization, Kochanski et al. (2025:3) define localization as consisting of “movements or shifts of power and agency among global and local actors that contribute to the transformation of local ownership and/or global norms and ideas.” Their definition captures the significance of power and agency, although their substantive focus remains confined almost exclusively to the interplay between global and local actors in world politics; and fails to acknowledge culture’s significance to the process.

The importance of culture is revealed in considerations about the scale of “local,” which might range from neighborhood to city or locale to region. As Tuan (1975) notes, the nation remains the largest unit that has been understood as local (as the expression “homeland” suggests). That might seem contrary to the widespread tendency to ascribe a self-evident quality to the nation and to delegate “the local” to sub-state units, while the nation itself is rarely understood as “local”. This is a relic of the methodological nationalism that used to be dominant in the past (cf. Martins, 1974: p. 276). In the 21st century, the reassertion of national populism is partly an expression of the newfound importance of locality in people’s lives (see Roudometof, 2025 for the full thesis).

It might also be relevant to add that the discipline of Sociology itself came to empirical inquiry in large part through the study of communal relations, and especially in addressing the perseverance of such relations in Western modernity. In the 21st century, failing to attend to the local inadvertently surrenders the concept to the lure of various localisms. To the extent that this is the case, the line that conceptually separates the condition of locality from the worldview of localism (further explored in later sections) is erased. In such a case, localism, a normative frame of reference, becomes indistinguishable from locality.

### 4.2 The conditions

The above processes generate three social conditions: globality, glocality, and locality. In past scholarship, globality has been defined as the condition generated by globalization’s growing “thickness.” Pieterse (2021) suggests that interconnectivity (a key indicator usually employed as a measure of globalization) is in fact a measure of globality. Interconnectivity describes the *effect* of globalization and not its causes. Irrespective of unanimity regarding which ought to be the more suited measurement, most scholars and laypersons accept that globality means the simultaneous and/or synchronous experience of events and relationships by the people of the globe, as well as the reflexivity generated by the sheer awareness of sharing such experiences with the rest of humanity. The COVID-19 pandemic has offered perhaps the most widely experienced example in recent memory.

Globality is experienced through the awareness of the physically absent but communicatively present other. This leads to simultaneity, e.g., doing things “together” at the same time while physically apart from each other. Synchronous comparison, that is, using contemporaries’ experience to formulate projects, policies, strategies, and so on becomes routinized; and historians have explored global interconnected histories for the years 1688, 1800, and 1979 (Conrad, 2017: p. 153). Simultaneity and synchronicity pose challenges for comparativists; their presence negates the researcher’s inclination to consider specific cases as truly independent of each other. For example, the sheer awareness and experience of the 1776 or the 1789 or the 1917 revolutions contributed to their employment as a reference point (more in Bayly, 2004; Johnson, 1991).

Decoding global events does not necessarily negate or obliterate local lenses to interpret an event or determine the local level of participation. On the contrary, local perception of the global is often determined by agency: TV coverage of the Olympic games offers a regular example, whereby stations cater to their national audiences by paying close attention to the athletes representing each nation, often to the detriment of adequately covering the games. It is precisely the realization that even so-called “global events” are decoded through very different lenses that leads to the notion of glocality. Glocality is the condition of experiencing the global locally, or through local lenses (which include power differentials, geopolitical and geographical factors, cultural distinctiveness, and so on). In this regard, most global events have a highly relevant glocal dimension.

To put it differently, events might be global in terms of scope or intent, but perception and reception are most often glocal. Glocality involves the construction and proliferation of a highly specific sub-type of hybridity, one that involves the merger of global and local (for a discussion, see Khondker, 2005; Roudometof, 2016: p. 13–16). In most instances, glocality contributes to the appearance of cultural pluralism in everyday life; but perhaps its main feature might be that *pastiche* or *bricolage* become routinized. The above paves the way for distinguishing between glocality and locality. Locality is also partly the result of cross-cultural influences, for practically all the world’s cultures have been intertwined over humanity’s history. But the cumulative influences of the Other do not necessitate the erosion of distinctiveness. On the contrary, locality suggests that the global influences that pervade the cultural life of a community, a place, or a country might *not* lead to mere *pastiche* or bricolage. Rather, it is possible for the local to acquire or have the ability and power to decode, deconstruct, absorb, and reconfigure such influences, to use their elements creatively and imaginatively within its hierarchical structures and consequently, to produce its own distinctiveness. This is not “isolated” from the global, but rather a partial “product” of global interconnectedness (as Appadurai, 1995 argues).

Locality therefore has a positive association with power; it is the flipside of the “global” and not its essentialist rival (Korff, 2003). For example, post-modern metropolitan regions are not mere agglomerations of previously distinct cultures, but configurations of new hybrid cultures (see Garcia Calcini, 1995; Smith, 2001). Despite global interconnectivity, De Blij (2009) demonstrates how the places people live have a strong impact upon their life chances and opportunities. Communities oriented or defined around places remain the norm or are revitalized, often through information-communication technology (for discussion and further examples, see Roudometof, 2023). That leads to the strengthening of locality instead of its disappearance.

In order to clearly differentiate between glocality and locality, it is necessary to acknowledge—as Bhabha (1994) and Burke (2009) do—that hybridity is not a distinct feature of modernity but an omnipresent feature of human cultures. Glocality and locality are differentiated on the basis of how such hybridity is understood and decoded by the actors themselves. When cross-cultural influences merge and fuse into a new identifiable blueprint and actors understand such influences as part of their own cultural universe the overall blueprint becomes that of a new locality. In contrast, when *pastiche* or *bricolage* prevail in the actors’ practices, but no identifiable new cultural pattern emerges the overall blueprint is that of glocality^10^.

### 4.3 The worldviews

Worldviews are typically prescriptive; they signify moral projects for the future, as well as visions about the world as it is going to (or should) be. These are means for opening the space of social and political imagination to the possibilities of the future. Debates that contemplate this “speculative” or normative understanding of globalization take place in a space of visions and projects. It is in this space that the three -isms of globalism, localism, and glocalism are located. Each proposes a moral vision of the future, a (positively or negatively viewed) prediction about the organization of the globe, and criteria for public policy. Globalism (Beck, 2000: p. 9) or what became more widely known as the post-1989 global neo-liberalism views the articulation of globality through very specific lenses in terms of its advocacy of free markets, relatively minimum state control over corporate activities, strong support for global trade, and an enduring belief in the value of the post-World War II geopolitical order.

Its archnemesis is localism, which rests on the proposition that globalism is detrimental to people’s welfare and ought to be reversed. Although often depicted as mere negation of globalism, localism stands for a different approach toward communal development (Wills, 2016). While in the United Kingdom localism has been instituted through political de-evolution, in the United States there is a long tradition of mistrust vis-à-vis the federal government. But while the relationship between localism and national politics may vary depending on context, localism is a vision of communal interdependence that actively discourages or counters global economic integration to protect local life-worlds and places by means of a communitarian outlook (for example, the “slow food” movement) (more in Maffesoli, 2021). Massey’s (2005: p. 5) statement is telling: “In the context of a world, which is…increasingly interconnected the notion of place (usually evoked as “local place”) has come to have totemic resonance.” This perseverance or even effervescence of localism frequently centers on the “nation” (which is the largest unit of the local). It is thus expressed as nationalist localism and often labeled “national populism” in the literature (for the full argument see Roudometof, 2025). Pursued by a multitude of political actors localism reverses dogmatic “one-worldism.” The juxtaposition between the two is a *leitmotif* in numerous debates in public policy and journalism on a broad array of topics ranging from democracy to economic development to immigration to economic inequality (and many other areas).

Lastly, glocalism is a blend of the other two that suggests flexibility to minimize costs and maximize benefits of the other two worldviews. Far less popular in the West, it is a strategy often employed in Southeast Asia; Singapore might be the most widely known example. Taking stock of cultural changes in 21st century China, Wang (2015: p. 2062) argues that “it is the “glocalising” orientation that is enabling China to relocate itself in the broader context of global culture.” China’s post-1979 developmental strategy is an iconic example of glocalism in public policy (see Changhe, 2011). By combining local and global, interconnectedness no longer leads to global conformity. China’s rules about foreign investment, for example, illustrate how state policy can be informed by a glocalist mentality. But using glocalism as a cultural shield against global encroachment is not necessarily dependent upon state policy. In Japan and India, for example, glocalism connects to deep cultural roots and does not rely exclusively on public policy. To be viable in such a milieu, brands adopt glocalist strategies—Shanghai Disneyland provides a relevant example (Sung, 2021; Roudometof, 2016: p. 102–113).

The three -isms (globalism, localism, and glocalism) are orientations that transcend the specificities of research areas or fields of study and shape attitudes at large. They play a key role both in the public’s mind as well as in shaping the worldviews of journalists, politicians, and opinion-makers. Even a cursory glance at the popular press and scholarly work shows that public intellectuals and academics are compelled to adopt a specific stance—be it positive or negative—vis-à-vis these worldviews, thereby blending descriptive and normative dimensions into their theses. In policymaking, these -isms shape specific projects or ideas of normative nature. While arguments have been made in favor of normative sociologies that could or should adopt (each of these three) normative stances, the extent to which such sociologies could successfully navigate the broader theme of normativity is quite debatable (see Abbott, 2018; Hammersley, 2024).

## 5 Conclusion

In this discussion, the objective of decentering globalization has been approached in terms of conceiving the global outside of the grand narrative of global modernization. While arguments concerning the pluralization of various life-worlds offer meaningful alternatives to global neoliberalism, these maintain a unitary conception of a socially integrated “world.” Thus, these are at odds with the multiple temporal frames and different thematic timelines that have emerged out of globalization scholarship. Breaking with past consensus, the approach adopted here accepts that no unitary grand narrative might be possible. Instead, an analytical framework is pursued that can be of relevance to varying degrees for different topics and/or genres of research.

As a result, the hitherto unitary conception of global modernization is pluralized and sub-divided into a trinity of terms—“global,” “local,” and “glocal.” In this discussion, each has been described as a process, a condition, and a worldview. The objective is to demonstrate that these should be viewed on an equal footing. None ought to be seen as normatively or analytically superior or dominant. Of particular importance is the implicit suggestion that successfully analyzing social relationships requires the consideration of global, glocal, and local as normative perspectives that are constitutive of the “real world.” When used as normative lenses in policy-making arguments, these worldviews invariably yield partial (some would say, one-sided) accounts of social realities. Irrespective of whether that is congruent with sociological practice, it is of critical importance to recognize the significance of these worldviews for research agendas and scholarship.

It is obviously impossible to address all possible theoretical threads within the confines of a single article. Suffice to say, the issue of power asymmetries among the proposed triad has been consciously left open to interpretation. The author’s objective has been to offer a preview or a snapshot of a broader framework. While global, glocal, and local offer partial and complementary accounts of social and cultural realities they further serve as normative worldviews for analysts and commentators. To grasp these concepts requires clear and unambiguous vocabulary; and this seemingly modest objective has guided this inquiry. Ultimately, no *ex-cathedra* statement can force the standardization of terms and concepts. By presenting the full range of theoretically available options, the preceding discussion has outlined a tool-kit of options to guide social-scientific practice on these concepts’ employment. To maximize portability the author has left upon the researchers to fix or standardize the power dynamics among local, glocal and global. This solution maximizes positionality and enables researchers to decode power dynamics according to their own general presuppositions or ideological orientations (see Alexander, 1988: p. 7).

## Footnotes

1 Giddens (1990: p. 1) and Alexander (2007) consider “globalization” as a term that denotes “global modernization.” See Wagner (2012) for a brief synopsis and Rosa (2013) for the most recent comprehensive interpretation.

2 Specifically, glocalization is derived from the Japanese dochakuka (see Edgington and Hayter, 2012) and hence it has non-Western origins. Unfortunately, many scholars in the West still adhere to the false claim that this concept’s roots lie in Western marketing (see Roudometof, 2016: p. 2–5).

3 While inclusion of Southern scholarship is an admirable goal, Go (2023) argues that moral worth should not be uncritically granted to scholars from specific regions. Following this recommendation, the discussion engages with those non-Western arguments directly linked with or responding to prior literature.

4 “World-making” practices are not necessarily surrendered to imperial blueprints, De Pina-Cabral (2017) argues. Instead, “it is up to us today to invent modes of carrying out our ethnography that correspond better to our post-imperial, globalized condition” (De Pina-Cabral, 2017: p. 185).

5 The notion of a “world” has been further extended into an argument whereby multiple such “worlds” co-exist within the globe. It has thus become the edifice upon which the pluriverse thesis has been erected (Escobar, 2020; Reiter, 2018). The pluriverse thesis argues for a plurality of such “worlds” and rejects the singularity associated with post-1989 “one-worldism.” It is not possible to fully address the pluriverse thesis here.

6 Conrad identifies five popular motors of social change in global history (technology, empire, economy, culture, and biology), arguing that “integration has multiple causes, multiple manifestations or consequences and…multiple chronologies” (Conrad, 2017: p. 103; see also Pieterse, 2021: pp. 65–84).

7 To eschew the goal of generating yet another master narrative of social change might seem like heresy, but that is so only to the extent that “theory” is erroneously equated with a master narrative of social change (for a discussion, see Abend, 2008).

8 In Robertson’s (1992) definition, globalization refers to “the compression of the world” and has a close affinity with the francophone mondialization (Nancy, 2007). In English it makes more sense to speak of “world-making.”

9 In response to a reviewer’s query, the tripartite division is not set within a multi-scalar hierarchy. The conceptualization of such hierarchies is predicated on the notion of absolute or geographical space instead of social space (see Roudometof, 2016: p. 31–38 for detailed discussion and comparison of these viewpoints).

10 For some examples of empirical research that illustrates these concepts’ operationalization, see Barber (2013) on how to grasp glocality within urban context; and Bampilis (2013) on how a commodity like whisky became emblematic of the culture of modernity in post-World War II Greece.
